# Risk factors for pericardial effusion after chemoradiotherapy for thoracic esophageal cancer—comparison of four-field technique and traditional two opposed fields technique

**DOI:** 10.1093/jrr/rry029

**Published:** 2018-04-11

**Authors:** Noriko Takata, Masaaki Kataoka, Yasushi Hamamoto, Shintaro Tsuruoka, Hiromitsu Kanzaki, Kotaro Uwatsu, Kei Nagasaki, Teruhito Mochizuki

**Affiliations:** 1 Department of Radiotherapy, Shikoku Cancer Center Hospital, Kou 160, Minami-Umemoto, Matsuyama, Ehime 791-0280, Japan; 2 Department of Radiology, Ehime University Hospital, Shitsukawa, Tohon, Ehime 791-0295, Japan

**Keywords:** esophageal cancer, radiotherapy, cardiac toxicity, pericardial effusion, risk factor

## Abstract

Pericardial effusion is an important late toxicity after concurrent chemoradiotherapy (CCRT) for locally advanced esophageal cancer. We investigated the clinical and dosimetric factors that were related to pericardial effusion among patients with thoracic esophageal cancer who were treated with definitive CCRT using the two opposed fields technique (TFT) or the four-field technique (FFT), as well as the effectiveness of FFT. During 2007–2015, 169 patients with middle and/or lower thoracic esophageal cancer received definitive CCRT, and 94 patients were evaluable (51 FFT cases and 43 TFT cases). Pericardial effusion was observed in 74 patients (79%) and appeared at 1–18.5 months (median: 5.25 months) after CCRT. The 1-year incidences of pericardial effusions were 73.2% and 76.7% in the FFT and TFT groups, respectively (*P* = 0.6395). The mean doses to the pericardium were 28.6 Gy and 31.8 Gy in the FFT and TFT groups, respectively (*P* = 0.0259), and the V40 Gy proportions were 33.5% and 48.2% in the FFT and TFT groups, respectively (*P* < 0.0001). Grade 3 pericardial effusion was not observed in patients with a pericardial V40 Gy of <40%, or in patients who were treated using the FFT. Although the mean pericardial dose and V40 Gy in the FFT group were smaller than those in the TFT group, the incidences of pericardial effusion after CCRT were similar in both groups. As symptomatic pericardial effusion was not observed in patients with a pericardial V40 Gy of <40% or in the FFT group, it appears that FFT with a V40 Gy of <40% could help minimize symptomatic pericardial effusion.

## INTRODUCTION

Esophageal cancer is the eighth most common malignancy in the world, and the sixth most common cause of cancer-related death [1]. Concurrent chemoradiotherapy (CCRT) is a standard treatment for locally advanced esophageal cancer in patients with a relatively good general condition. However, long-term heart and lung toxicity has been reported after definitive chemoradiotherapy for thoracic esophageal cancer [2]. The cardiac adverse events include pericarditis/pericardial effusion, myocardial injury, vascular stenosis, valvar injury, and/or conduction abnormalities. Acute cardiac injury often manifests as pericarditis and is usually transient, although it can also be chronic [3]. Subacute/delayed pericardial disease can occur at intervals of months to years after radiotherapy, and includes pericarditis and chronic pericardial effusion, which is usually asymptomatic. Although most cases resolve spontaneously, ~20% progress to chronic and/or constrictive pericarditis [4].

The traditional irradiation technique for thoracic esophageal cancer is the two opposed fields technique (TFT). However, in an attempt to minimize cardiac adverse events, the four-field technique (FFT) (two opposed anteroposterior fields combined with two opposed oblique fields) has become frequently used in clinical practice. This is because FFT is a simple technique for reducing the heart volume (especially the left ventricle) that receives high doses, compared with the TFT, although it is unclear whether FFT can decrease the incidence of pericardial effusion. Therefore, this retrospective study evaluated dose–volume histogram (DVH) data for the pericardium to determine whether FFT decreased the incidence of pericardial effusion, compared with TFT, among patients who received definitive CCRT for thoracic esophageal cancer.

## MATERIALS AND METHODS

### Patients

Between January 2007 and June 2015, 169 patients with newly diagnosed middle and/or lower thoracic esophageal cancer received definitive CCRT in one institution. This study’s retrospective protocol was approved by our institutional review board (No. 27 in 2016). Patients were excluded if they did not undergo follow-up computed tomography (CT) after more than 6 months from the end of the CCRT (*n* = 46), or if the initial treatment plan involved the three-field technique (*n* = 1). In addition, patients in whom the imaging range of planning CT was narrow and the whole pericardium was not imaged were excluded because DVH analysis of the pericardium could not be performed (*n* = 28). Thus, a total of 94 patients were assigned to the FFT group (*n* = 51) or the TFT group (*n* = 43) according to their initial treatment plan. Tumors were staged according to the 7th version of the *UICC-AJCC TNM Staging Manual*, and based on the findings from fluoro-D-glucose positron emission tomography (FDG-PET)/CT or contrast-enhanced CT.

### Treatment

Treatment plans were created using the Eclipse planning system (Varian Medical Systems, Palo Alto, CA), and 3-mm CT slices that were acquired during free breathing (HiSpeed NX/i Smart Gantry system; General Electric Healthcare, Little Chalfont, UK). The gross tumor volume (GTV) was defined as the primary tumor plus any metastatic lymph nodes. Primary tumors were identified based on the CT, FDG-PET/CT, and/or endoscopy findings. If the primary tumor could not be identified on the CT and/or FDG-PET/CT images, metallic clips were placed on the esophageal wall on the oral and anal sides of the primary tumor during the endoscopy before the CT simulation. Metastatic lymph nodes were also identified using the CT and/or FDG-PET/CT images. The clinical target volume (CTV) for the primary tumor was defined as the tumor’s volume plus margins of 2–3 cm in the craniocaudal direction and 0.5 cm in the other directions. The nodal CTV was defined as the volume of any metastatic lymph nodes plus margins of 0.5 cm in all directions. The overall CTV was defined as the primary and nodal CTVs, although prophylactic treatments [elective nodal irradiation (ENI)] were also included in the CTV for some patients, based on the physician’s discretion. The prophylactic treatments target regions where lesions might be present. The planning target volume (PTV) was defined as the overall CTV plus margins of 0.5 cm in all directions.

The patients had received a dose of 60 Gy in 30 fractions over 6 weeks using 10-MV X-rays from a linear accelerator (Clinac 21-EX; Varian Medical Systems). The first 40 Gy were delivered to the PTV using either TFT or FFT (initial plan), and the remaining 20 Gy were administered using off-cord boost plans in principle. When off-cord boost plans were made, CT simulation was newly performed. In off-cord boost plans, the spinal cord and any prophylactic regions were excluded from the radiation fields, and the final 20 Gy were delivered to the reduced PTV. Some of the patients who were treated using FFT did not need a change to an off-cord plan because their initial plans were able to keep the dose of the spinal cord below 40 Gy while administering 60 Gy to the PTV. The chemotherapy regimens were based on cisplatin (70 mg/m^2^ on Days 1 and 29) and 5-fluorouracil (700 mg/m^2^ on Days 1–4 and 29–32), although the doses were decreased if necessary. All patients received concurrent chemoradiotherapy as the initial treatment.

### Dosimetric analysis

The pericardium was defined as the sac-like structure (thickness: 3 mm) at the surface of the heart and the roots of the great vessels. The entire pericardial volume was contoured on the treatment planning CT images according to the RTOG Contouring Atlases for Organs at Risk in Thoracic Radiation Therapy [5]. The pericardial DVH was subsequently generated using the treatment planning system. The pericardium was contoured both on CT images for initial plans and on CT images for boost plans. Using image-fusion function by rigid image registration of the treatment planning system, CT images for the boost plan were superimposed on CT images for the initial plan. Then, we made the sum-plan, including the initial plan and the boost plan on CT images for the initial plan. DVH analysis was performed using the sum-plan. The percent volumes that received doses of ≥5 Gy, ≥10 Gy, ≥20 Gy, ≥30 Gy, ≥40 Gy, ≥50 Gy and ≥60 Gy were expressed as V5 Gy, V10 Gy, V20 Gy, V30 Gy, V40 Gy, V50 Gy and V60 Gy, respectively.

### Evaluation of pericardial effusion and follow-up

In principle, follow-up CT was performed every 2–3 months during the first 3 years and then every 2–6 months thereafter. The time to the appearance of pericardial effusion was calculated from the beginning of CCRT. All patients were followed-up using CT for ≥6 months. Grading of pericardial effusion was performed according to the Common Terminology Criteria for Adverse Events (version 4.0): Grade 0 = no pericardial effusion, Grade 2 = asymptomatic small-to-moderately sized effusion, Grade 3 = effusion with physiological symptoms, Grade 4 = effusion with life-threatening symptoms that required urgent intervention, and Grade 5 = death.

### Statistical analysis

The cumulative incidences of pericardial effusion were compared using the Kaplan–Meier method and the log-rank test. Differences in the V5 Gy, V10 Gy, V20 Gy, V30 Gy, V40 Gy, V50 Gy and V60 Gy values were evaluated using the Wilcoxon test. Logistic regression analyses were used to investigate the risk factors for pericardial effusion. However, as some factors could confound each other, we selected optimal factors using stepwise regression analysis (a combination of forward selection and backward elimination). The optimal risk factors were subsequently used in logistic regression analysis. Statistical analyses were performed using JMP software (version 12.0; SAS Institute Inc., Cary, NC).

## RESULTS

**Fig. 1.**
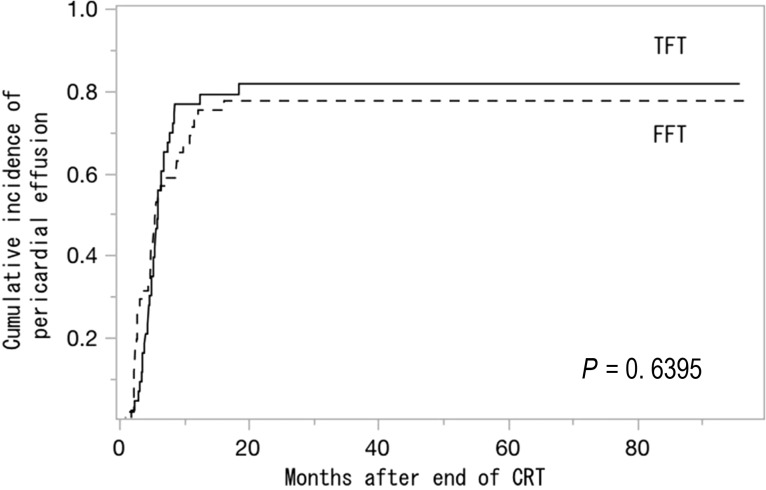
Cumulative incidence of pericardial effusion. TFT = two opposed fields technique, FFT = four-field technique, CRT = chemoradiotherapy.

The median patient age was 67 years (range: 38–87 years), and the male/female ratio was 84/10 (Table 1). Some differences were found between the FFT and TFT groups, with early-stage cancer being more frequent in the FFT group, and the initial field length tending to be longer in the TFT group. The overall follow-up periods ranged from 6.7 months to 96.6 months (median: 33.9 months). Pericardial effusion was observed in 74 patients (79%), and appeared at 1–18.5 months (median: 5.3 months) after the CCRT. The cumulative incidences of pericardial effusion in the FFT group were 73% at 1 year, 78% at 2 years and 78% at 3 years. These rates were not significantly different, compared with the rates in the TFT group (77%, 82% and 82%, respectively; *P* = 0.6395). The cumulative incidence of pericardial effusion is shown in Fig. 1. Of the 74 patients with pericardial effusion, 32 (31 Grade 2 and one Grade 3) had recurrence before or after the onset of pericardial effusion or simultaneously. A total of 23 patients with Grade 2 pericardial effusion experienced recurrence after the appearance of the pericardial effusion, which was considered to be unrelated to the recurrence or salvage therapy. Seven patients with Grade 2 pericardial effusion suffered from recurrence at the same time as appearance of pericardial effusion. Because the pericardial effusion decreased and remained small in size in follow-up CT, these pericardial effusions were diagnosed as being unrelated to the recurrence. In one patient with Grade 2 pericardial effusion, the pericardial effusion appeared 5 months after the recurrence in the gastric lymph node. Because the recurrence site was outside the first CCRT field, it was treated with salvage CCRT. The pericardial effusion finally disappeared. One patient with Grade 3 pericardial effusion recurred 5 months after the pericardial effusion. In this patient, pericardial drainage was performed 16 months after the appearance of pericardial effusion, and the result of the cytologic examination was negative. For these reasons, the cases of pericardial effusion in this study were considered unrelated to the recurrence or salvage therapy.

**Table 1.** Patient characteristics according to treatment techniques

		TFT (*n* = 43)		FFT (*n* = 51)		*P* value
Age (years)	Median	68	(38–87)	66	(45–84)	0.1247
Sex	Male	39	91%	45	88%	0.6997
	Female	4	9%	6	12%	
Tumor involving lower thoracic esophagus	Yes	18	42%	18	35%	0.5141
	No	25	58%	33	65%	
T stage	1	6	14%	29	57%	0.0002
	2	18	42%	12	24%	
	3	13	30%	9	19%	
	4	6	14%	1	2%	
N stage	0	16	37%	36	71%	0.0032
	1	10	23%	10	20%	
	2	15	35%	4	8%	
	3	2	5%	1	2%	
Clinical stage	1	13	30%	36	71%	0.0012
	2	8	19%	4	8%	
	3	21	49%	10	20%	
	4 (M1 LYM)	1	2%	1	2%	
Field length	<20 cm	13	30%	25	49%	0.0644
	≧20 cm	30	70%	26	51%	

TFT = two field technique, FFT = four-field technique.

Table 2 shows the results of the DVH analysis according to the treatment techniques. The mean pericardial doses were 31.8 Gy and 28.6 Gy for the FFT and TFT groups, respectively. The pericardial V40 Gy and mean dose in the FFT group were significantly smaller than those in the TFT group (*P* < 0.0001 and *P* = 0.0259, respectively). However, no significant differences were observed when the V5 Gy, V10 Gy, V20 Gy, V30 Gy, V50 Gy and V60 Gy values were compared between the two groups.

**Table 2.** DVH parameters according to treatment techniques

Dosimetric factors	TFT (*n* = 43)	FFT (*n* = 51)	*P* value
V5 Gy (%)	77.2	74.6	0.4616
V10 Gy (%)	72.3	62.4	0.3038
V20 Gy (%)	65.9	61.1	0.2646
V30 Gy (%)	57.1	52.7	0.1451
V40 Gy (%)	48.2	33.5	<0.0001
V50 Gy (%)	25.7	22.8	0.0757
V60 Gy (%)	7.9	7.8	0.8734
Mean Dose (Gy)	31.8	28.6	0.0259

TFT = two field technique, FFT = four-field technique.

Grade 3 pericardial effusion was observed in the TFT group (7%, 3/43), but was not observed in the FFT group (0/51; *P* = 0.0921, Fisher’s exact test). No cases of Grade 4 or 5 pericardial effusion were identified. The median time from the beginning of CCRT to pericardial drainage in the patients with Grade 3 pericardial effusion was 23 months (22 months, 23 months and 25 months), and the V40 Gy values in these patients were 40.3%, 47.6% and 78.8%, respectively. Grade 3 pericardial effusion was not observed in patients with a pericardial V40 Gy of <40%.

Table 3a and b shows the univariate analyses of clinical factors that were related to all-grade and Grade 3 pericardial effusion. Significant differences were observed in field length and tumor localization in the lower esophagus for all-grade pericardial effusion. Grade 3 pericardial effusion was significantly associated with TFT (*P* = 0.0283). Table 4a and b shows the univariate analyses of dosimetric factors. Significant differences were observed in all dosimetric parameters between patients with and without pericardial effusion, although only V40 Gy was significantly associated with Grade 3 effusion. In the multivariate analysis of pericardial effusion, stepwise regression analysis with a cut-off *P* value of 0.25 was performed including all clinical and DVH factors, because we did not know which factors were related to pericardial effusion. The results revealed that two factors, involving lower esophagus and V20 Gy, were picked-up for evaluation. In the multivariate analysis, only V20 Gy was a significant risk factor for Grade 2–3 pericardial effusion (*P* = 0.001) (Table 5). Multivariate analysis was not performed for symptomatic Grade 3 pericardial effusion based on the limited number of cases.

**Table 3a.** Univariate analysis of clinical factors influencing all Grade pericardial effusion

Clinical factors		Grade 2, 3 (*n* = 74) (%)	Grade 0 (*n* = 20) (%)	Odds ratio	95% CI	*P* value
Age (<65 vs >65)	<65	35	40	1.2	0.4–3.4	0.6894
Sex (male vs female)	male	88	95	2.6	0.5–50.1	0.3201
T stage (1, 2 vs 3, 4)	1, 2	66	80	2.0	0.7–7.7	0.2222
N stage (0, 1 vs 2, 3)	0, 1	76	80	1.3	0.4–4.9	0.6813
Clinical stage (1, 2 vs 3, 4)	1, 2	64	70	1.3	0.5–4.2	0.5863
Involving lower esophagus (no vs yes)	No	57	80	3.1	1.0–11.4	0.0492
Field technique (TFT vs FFT)	TFT	47	40	0.7	0.3–2.0	0.5598
Field length (<20 cm vs >20 cm)	<20 cm	34	65	3.6	1.3–10.8	0.0122

TFT = two field technique, FFT = four-field technique.

**Table 3b.** Univariate analysis of clinical factors influencing Grade 3 pericardial effusion

Clinical factors		Grade 3 (*n* = 3) (%)	Grade 0, 2 (*n* = 91) (%)	Odds ratio	95% CI	*P* value
Age (<65 vs >65)	<65	33	36	1.1	0.1–25.0	0.9167
Sex (male vs female)	male	100	89	2.7E-08	0–7.9	0.4073
T stage (1, 2 vs 3, 4)	1, 2	33	70	4.7	0.4–104.5	0.1946
N Stage (0, 1 vs 2, 3)	0, 1	33	78	7.1	0.6–157.3	0.1050
Clinical stage (1, 2 vs 3, 4)	1, 2	33	66	3.9	0.4–85.2	0.2586
Involving lower esophagus (no vs yes)	No	67	62	0.8	0.04–8.7	0.8561
Field technique (TFT vs FFT)	TFT	100	44	1.2E-08	0–0.7	0.0283
Field length (<20 cm vs >20 cm)	<20 cm	67	41	1.4	0.1–30.1	0.7970

TFT = two field technique, FFT = four-field technique.

**Table 4a.** Univariate analysis of dosimetric factors influencing all Grade pericardial effusion

	Median pericardial volume		Univariate analysis		
Parameters	Grade 2, 3	Grade 0	Odds ratio^a^	95% CI	*P* value
V5 Gy (%)	79.7 (51.8–100.0)	66.5 (45.7–88.5)	1.08	1.03–1.13	0.0004
V10 Gy (%)	74.2 (46.5–97.6)	61.1 (40.8–84.7)	1.08	1.03–1.13	0.0003
V20 Gy (%)	68.2 (38.8–88.2)	53.4 (33–80.2)	1.08	1.03–1.13	0.0003
V30 Gy (%)	55.9 (32.1–83.3)	44.5 (19.5–74.3)	1.08	1.03–1.13	0.0005
V40 Gy (%)	43.8 (19.2–78.8)	34.2 (8.9–57.1)	1.06	1.02–1.11	0.0024
V50 Gy (%)	24.5 (10.9–47.3)	16.5 (6.7–35.8)	1.13	1.05–1.23	0.0009
V60 Gy (%)	8.0 (0.3–21.6)	4.5 (0.4–14.8)	1.19	1.04–1.39	0.0228
Mean dose (Gy)	31.7 (16.7–44.1)	25.6 (14.3–37.9)	1.16	1.06–1.28	0.0005

^a^Odds ratio was the value per unit increase.

**Table 4b.** Univariate analysis of dosimetric factors influencing Grade 3 pericardial effusion

Parameters	Grade 3	Grade 0, 2	Odds ratio^a^	95% CI	*P* value
V5 Gy (%)	79.5 (63.4–100.0)	77.2 (45.7–95.4)	1.04	0.94–1.19	0.4344
V10 Gy (%)	75.2 (58.0–97.6)	71.9 (40.8–91.3)	1.05	0.95–1.19	0.3354
V20 Gy (%)	69.4 (52.8–86.9)	65.7 (33–88.2)	1.04	0.95–1.18	0.4110
V30 Gy (%)	57.4 (49.2–83.3)	53.9 (19.5–79.7)	1.06	0.97–1.18	0.2365
V40 Gy (%)	47.6 (40.3–78.8)	40.7 (8.9–69.5)	1.09	1.00–1.21	0.0480
V50 Gy (%)	26.7 (21.1–41.0)	22.8 (6.7–47.3)	1.07	0.94–1.22	1.0700
V60 Gy (%)	6.6 (4.7–11.1)	7.3 (0.3–21.6)	0.98	0.71–1.24	0.8738
Mean dose (Gy)	32.4 (26.5–44.1)	30.20 (14.3–41.9)	1.12	0.93–1.42	0.2288

^a^Odds ratio was the value per unit increase.

**Table 5.** Multivariate analysis for all grades pericardial effusion

Factors	Odds ratio	95% CI	*P* value
Involving lower esophagus (no vs yes)	2.12	0.63–8.45	0.2451
V20 Gy	1.07^a^	1.03–1.13	0.0021

^a^Odds ratio was the value per unit increase.

## DISCUSSION

Compared with radiotherapy alone, CCRT is a standard treatment for locally advanced or unresectable esophageal cancer and provides superior survival and local control [6]. Late cardiopulmonary toxicities have recently been recognized [2], although there is no clear quantitative dose and/or volume dependence for most cardiac toxicity. No previous data are available for comparing dose volumes between FFT and TFT for esophageal cancer, although FFT is a simple irradiation technique that is thought to decrease the heart volume that receives high doses. When we compared FFT and TFT, we found that the V40 Gy and mean dose values in the FFT group were significantly smaller than those in the TFT group. Nevertheless, there was selection bias in our sample, as the FFT group included more patients with early clinical stage, while the TFT group had a longer mean initial field length (22.8 cm vs 20.8 cm). If this difference in field length was substantial, we would expect noticeable differences in all DVH parameters, including the low-dose spectrum. Thus, it appears that the differences in the V40 Gy and mean dose values were related to other factors, including the field techniques. However, FFT did not decrease the incidence of all-grade pericardial effusion, compared with TFT, although TFT was a significant risk factor for symptomatic pericardial effusion (i.e. Grade 3). Based on these data, it appears that FFT generates low pericardial V40 Gy and mean dose values, which might limit the risk of symptomatic pericardial effusion.

The univariate analyses revealed that field length and tumor localization involving the lower esophagus were significant risk factors for all-grade pericardial effusion, although these factors were not significant for symptomatic pericardial effusion (Table 3). In this context, field length is a known risk factor for pericardial effusion, as Fukada *et al.* reported that a field length of >20 cm was a significant risk factor for the development of pericardial effusion [7]. Moreover, the field length can vary if the patient is treated using ENI or involved field irradiation (IFRT), although there is no clear consensus regarding the role of ENI. Some researchers have suggested that IFRT could be superior to ENI, as it can minimize adverse effects without increasing locoregional failure [8], while other researchers have reported that ENI was effective for preventing regional nodal failure [9]. Although a prospective trial would clarify the survival benefit of ENI, radiotherapy using smaller fields (e.g. IFRT) may be effective for preventing pericardial effusion. Few reports have examined the relationship between tumor location and pericardial effusion, although it does not appear to be a risk factor for pericardial effusion [7, 10]. Thus, our findings from the multivariate analysis are consistent with those results.

Some authors have reported that dose–volume factors predict the development of pericardial effusion. For example, Hayashi *et al.* reported that the most significant predictors of pericardial effusion after chemotherapy and radiotherapy were V5 Gy to V60 Gy of the heart [11]. In addition, Wei *et al.* have reported that V30 Gy of the pericardium/heart was the only independent risk factor for pericardial effusion after chemoradiotherapy [12]. Furthermore, Tamari *et al.* evaluated patients with Stage I esophageal cancer who received chemoradiotherapy, and reported that the cumulative rate of pericardial effusion was 52.2%, with pericardial V30 Gy of >41.6% being a significant risk factor [10]. Thus, the volume of the heart/pericardium receiving relatively low doses (i.e. 10–30 Gy) is likely associated with the development of pericardial effusion. In the present study, V20 Gy was a significant risk factor for pericardial effusion in the univariate and multivariate analyses, which suggests that a relatively low dose (e.g. V20 Gy) can be related to all-grade pericardial effusion.

In contrast, symptomatic pericardial effusion seems to be induced by slightly higher doses. For example, Fukada *et al.* reported that the pericardial V30 Gy–V45 Gy values were significantly higher in patients with symptomatic pericardial effusion, compared with patients with asymptomatic pericardial effusion [13]. Thus, they concluded that the mean pericardial dose was the strongest risk factor for symptomatic pericardial effusion, and that a mean pericardial dose of 36.5 Gy and a pericardial V45 Gy of 58% seemed to be the optimal cut-off values for predicting symptomatic pericardial effusion. Those findings suggest that relatively low doses (10–30 Gy) can induce asymptomatic pericardial effusion, but higher doses (~40 Gy) are needed to induce symptomatic pericardial effusion. The present study only included three patients with Grade 3 pericardial effusion, who could not be evaluated in the multivariate analysis, although these cases all involved TFT and had a pericardial V40 Gy of >40% (40.3%, 47.6% and 78.8%). Moreover, the V40 Gy value in the FFT group was significantly smaller than that in the TFT group, and V40 Gy was significantly associated with symptomatic pericardial effusion. Therefore, FFT appears to have provided a small V40 Gy and may be useful for minimizing the risk of symptomatic pericardial effusion.

The median time to the appearance of pericardial effusion was 5.3 months, with a trend towards a longer time for symptomatic pericardial effusion (~2 years) compared with asymptomatic pericardial effusion. Previous reports have also indicated that most symptomatic pericardial effusion is detected at >1 year [10, 13, 14], and that some cases may not be detected until 5 years after the CCRT [13]. Thus, the onset of symptomatic pericardial effusion seems to be relatively late, and we suspect that prolonged and careful follow-up is needed in order to detect this event, despite the limited number of patients who experience symptomatic pericardial effusion.

Previous studies have indicated that late toxicities involving the coronary artery, capillary myocardium, valves, and conducting system are decreased when smaller heart volumes receive high doses. For example, Ogino *et al.* reported that the risk of symptomatic cardiac disease (pericardial effusion, valvular disease, tachycardia, atrial fibrillations, coronary artery disease, and congestive heart failure) appeared to be related to relatively high-dose volumes (i.e. V45 Gy, V50 Gy and V55 Gy) [14]. In addition, a single photon emission computed tomography (SPECT) study revealed that myocardial fatty acid metabolism seemed to be affected by intermediate and high heart doses (40–60 Gy) [15]. Furthermore, a myocardial perfusion SPECT study revealed that the percent volume of the heart receiving intermediate doses (37–40 Gy) seemed to be associated with the development of new perfusion defects during radiation therapy [16]. These reports indicate that decreased cardiac volumes receiving intermediate/high radiation doses were associated with fewer late cardiac adverse events, although we only identified two cases involving non-effusion cardiac events (two cases of myocardial ischemia), which precluded further analysis.

The present study has several limitations. First, this was a retrospective study in a single institution with a limited sample size. Second, the limited number of patients with symptomatic pericardial effusion precluded a detailed statistical evaluation. Finally, late cardiac events that occurred after several years could not be evaluated. In addition, the influence of salvage chemoradiotherapy for recurrent disease and/or recurrent disease could not be evaluated in patients in whom tumor recurrence preceded the appearance of pericardial effusion. Only one patient experienced tumor recurrence in the upper abdomen preceding appearance of pericardial effusion, and that patient received salvage chemoradiotherapy. Because the pericardium was almost outside of the irradiation field of salvage chemoradiotherapy and distant from the recurrent site, the influence of the salvage irradiation and recurrent disease on the pericardium might have been small.

## CONCLUSION

To the best of our knowledge, this is the first report to indicate that, compared with TFT, FFT decreased the pericardial mean dose and V40 Gy values. Our findings also indicate that a pericardial V40 Gy of >40% may predict the development of symptomatic pericardial effusion. However, further studies are needed to clarify whether FFT can reduce symptomatic cardiac toxicity.
